# Mice humanized by syntenic replacement with full-length NLRP3 disease-associated variants model the clinical cryopyrinopathy continuum

**DOI:** 10.1172/jci.insight.194677

**Published:** 2026-03-09

**Authors:** John N. Snouwaert, MyTrang Nguyen, Christopher A. Gabel, Ivona Aksentijevich, Jenny P.-Y. Ting, Beverly H. Koller

**Affiliations:** 1 Department of Genetics, University of North Carolina at Chapel Hill, Chapel Hill, North Carolina, USA.; 2 NodThera, Seattle, Washington, USA.; 3 Inflammatory Disease Section, National Human Genome Research Institute, Bethesda, Maryland, USA.; 4 Lineberger Comprehensive Cancer Center;; 5 Department of Microbiology and Immunology, School of Medicine; and; 6 Center for Translational Immunology, University of North Carolina at Chapel Hill, Chapel Hill, North Carolina, USA.

**Keywords:** Genetics, Immunology, Inflammation, Genetic diseases, Innate immunity, Mouse models

## Abstract

Next-generation sequencing technologies are increasingly used to diagnose genetic disorders, particularly immunological diseases with broad and overlapping immune dysregulation. Cryopyrin-associated periodic syndromes (CAPS) are caused by gain-of-function mutations in NLRP3 and include 3 autoinflammatory diseases spanning a continuum of severity: familial cold autoinflammatory syndrome (FCAS), Muckle-Wells syndrome (MWS), and neonatal-onset multisystem inflammatory disease (NOMID). Linking NLRP3 variants to protein dysfunction and clinical phenotype remains challenging because of genetic modifiers and environmental factors. We report the generation and phenotyping of 5 mouse lines expressing either the common human *NLRP3* allele or 1 of 4 CAPS mutations spanning the disease spectrum from FCAS to NOMID. In these lines, the murine *Nlrp3* locus is replaced by syntenic integration of the human *NLRP3* locus, yielding 1 line with the common allele and 4 lines each carrying a distinct CAPS mutation. Unlike models in which a human mutation is introduced into the mouse protein, these lines recapitulate the spectrum of disease severity observed in humans. These findings support a model in which evaluation of nonsynonymous mutations in mice is optimized when introduced in the context of the human gene. This suggests that species-specific regulation and/or intramolecular epistasis may impact modeling of disease-associated variants.

## Abstract 2

Humanization of the entire Nlrp3 locus, including regulatory regions, with human disease-associated variants yields mouse models that mirror the human spectrum of cryopyrin-associated periodic syndromes.

## Graphical abstract

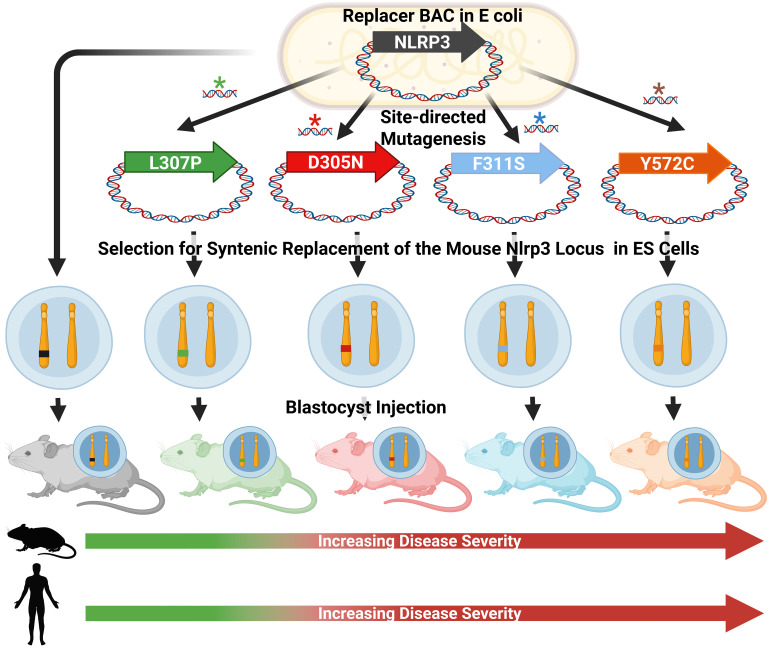

## Introduction

Familial cold autoinflammatory syndrome (FCAS) (1), Muckle-Wells syndrome (MWS) (2, 3), and neonatal-onset multisystem inflammatory disease (NOMID), also known as chronic infantile neurological cutaneous articular syndrome (CINCA) (4, 5), are rare autosomal dominant inheritance syndromes, which present with a spectrum of symptoms that include fever, elevated acute-phase responses, neutrophilic inflammation, urticaria-like rashes, and arthritis. FCAS presents with cold-induced fever and urticaria, while MWS may progress to systemic amyloidosis and hearing loss (6). NOMID/CINCA, the most severe form, often begins in the neonatal period or early infancy with pervasive multisystem inflammation, including CNS inflammation, potentially leading to early mortality in the absence of targeted therapies (5).

Seminal genetic studies of large multigenerational FCAS families identified a causal mutation in the gene initially called *cryopyrin*, now known as *NLRP3* (7). Subsequent work identified distinct missense mutations in the same gene in patients with MWS and NOMID/CINCA (8–10), establishing these disorders as a clinical continuum termed cryopyrin-associated periodic syndrome (CAPS or recently NLRP3-AID) (11). These discoveries accelerated understanding of NLRP3 function, particularly its role in IL-1β maturation and release (12), and highlighted the value of defining the genetic basis of inflammatory disease. Rapid genetic diagnosis soon became essential, as clinical studies demonstrated that off-label use of the IL-1β inhibitor anakinra — originally developed for septic shock (13) and later for rheumatoid arthritis (14) — was highly effective and lifesaving in patients with CAPS (15).

*NLRP3* is a member of the nucleotide-binding domain leucine-rich repeat gene family (16). Together with the adaptor apoptosis-associated speck-like protein containing a CARD (ASC) and pro–caspase-1, it forms the NLRP3 inflammasome (17), a multiprotein complex essential for innate immunity. Inflammasome activation leads to caspase-1 autoproteolysis, which in turn enables maturation of pro–IL-1β and pro–IL-18 and triggers pyroptosis (18). Gasdermin D, activated by caspase-1, forms membrane pores that drive cell lysis and cytokine release (19). CAPS-associated NLRP3 mutations are gain-of-function missense variants that alter NLRP3 structure and permit activation by subthreshold stimuli, shifting the protein from an autoinhibited to an active state. Recent studies support a model in which the CAPS mutations result in auto-active inflammasomes, either by stabilizing the active conformation or by destabilizing the inactive conformation of NLRP3 (20, 21). Regardless of the mechanism, inflammasome assembly and IL-1β release occur under conditions that would not normally lead to activation (22–24). In vitro monocyte assays and reconstitution systems have provided mechanistic insight into this process (21, 25). However, they often fail to distinguish between mutations tied to varying autoinflammation severities. This likely reflects the diversity of NLRP3-expressing myeloid lineages driving disease. A mechanistic understanding of how specific amino acid substitutions manifest as variability in disease severity remains incomplete.

Many CAPS-associated variants alter residues conserved between human and mouse, and mouse knockin models carrying these disease mutations have been widely used to study inflammasome-driven immune dysfunction (26–29). In addition, recently a porcine model carrying the NLRP3 R259W substitution, which corresponds to the human R260W Muckle-Wells mutation, has been generated using CRISPR/Cpf1 (30). Although the mouse models reproduce key inflammatory features, they have important limitations. Disease severity in knockin mice is generally far greater than that observed in human carriers, with lifespans ranging from less than 1 day to a maximum of about 45 weeks, and the relative severity of phenotypes does not align with that seen clinically (31). These discrepancies raise concerns regarding the use of knockin mice to model and compare phenotypes associated with CAPS-linked missense mutations.

Here, we describe the generation of CAPS mouse lines in which the entire mouse *Nlrp3* locus was replaced with the syntenic human segment carrying a single CAPS-associated *NLRP3* variant, ensuring that both *NLRP3* expression and protein are of human origin and that the mouse locus is absent. Using this strategy, we generated 4 lines that span the full spectrum of CAPS severity reported in patients (10). One line carries the L307P allele (rs180177431), identified in patients with FCAS and associated with mild disease (8, 32). A second line carries the D305N mutation (rs121908153), reported in both MWS (9, 33, 34) and NOMID/CINCA cases (10, 32, 35, 36). The third line carries the Y572C allele (rs180177438), one of the most common NOMID/CINCA mutations and associated with severe neurologic symptoms, including mental deficits and epilepsy (10). Finally, we generated a line carrying the F311S mutation (rs121908154), which has been identified in patients with NOMID/CINCA (35, 37, 38), although its relative severity compared with D305N and Y572C remains unclear because of the limited number of reported cases. As a control, we used a line in which the mouse *Nlrp3* locus was replaced by the common human allele (hNLRP3).

Evaluation of these 5 mouse lines shows that introducing CAPS mutations into the humanized locus produces models in which disease severity aligns closely with corresponding human phenotypes. These lines enable analysis of how specific NLRP3 variants affect development, longevity, and pathogenesis of chronic systemic and CNS inflammation. This approach can also support functional evaluation of new or rare variants of unknown significance detected through whole-exome sequencing and whole-genome sequencing and facilitate studies of NLRP3 regulatory mechanisms, which influence numerous diseases beyond CAPS. The models further allow assessment of environmental impacts on mutant inflammasome activity and, by extension, on the common *NLRP3* allele. More broadly, our findings suggest that modeling human disease–associated mutations in the context of the human protein and promoter may yield more accurate phenotypes than introducing the same mutations into the orthologous mouse gene.

## Results

### Generation of mouse lines carrying the L307P, F311S, and Y572C mutations.

The deletion of the entire mouse *Nlrp3* locus in embryonic stem (ES) cells and its reconstitution with the syntenic segment of either the common or D305N disease-associated human *NLRP3* locus has been previously described (39). An identical strategy was used for the generation of 3 additional ES cell lines in which the deleted mouse *Nlrp3* locus was replaced with a human *NLRP3* gene carrying the L307P, F311S, or Y572C mutation, respectively. The structure of the humanized *NLRP3* locus was verified using Southern blot and sequence analysis. The clinical phenotypes of the CAPS-associated missense mutations introduced into the mouse genome are summarized and compared in Supplemental Table 1; supplemental material available online with this article; https://doi.org/10.1172/jci.insight.194677DS1

To gain immediate insights into the expression and function of the mutant NLRP3 proteins, we carried out a single pilot experiment utilizing 5 ES cell lines: 4 unique lines, each engineered to carry a different CAPS-associated mutation, and 1 line carrying hNLRP3. These ES cells were differentiated into macrophages, then exposed to LPS, and IL-1β release was measured (Supplemental Figure 1A). As expected, LPS alone was sufficient to trigger IL-1β release from cells expressing mutant human NLRP3 but not from cells expressing the common human allele. Minimal IL-1β release was observed from the line carrying the mild L307P FCAS mutation. Release was intermediate for D305N, identified in both patients with NOMID and MWS. Markedly higher release was detected from cells carrying the F311S or Y572C mutations, which are associated with the most severe disease, NOMID/CINCA. Release of 2 LPS-responsive, NLRP3-independent cytokines, TNF-α and IL-6 (Supplemental Figure 1, B and C), was robust across genotypes. Given the differences in IL-1β release between lines and the alignment of this release with CAPS severity, mouse lines were generated from these ES cells to further investigate the genotype–phenotype relationship in vivo.

### Phenotyping mice heterozygous for CAPS-associated mutations.

**Figure 1:**
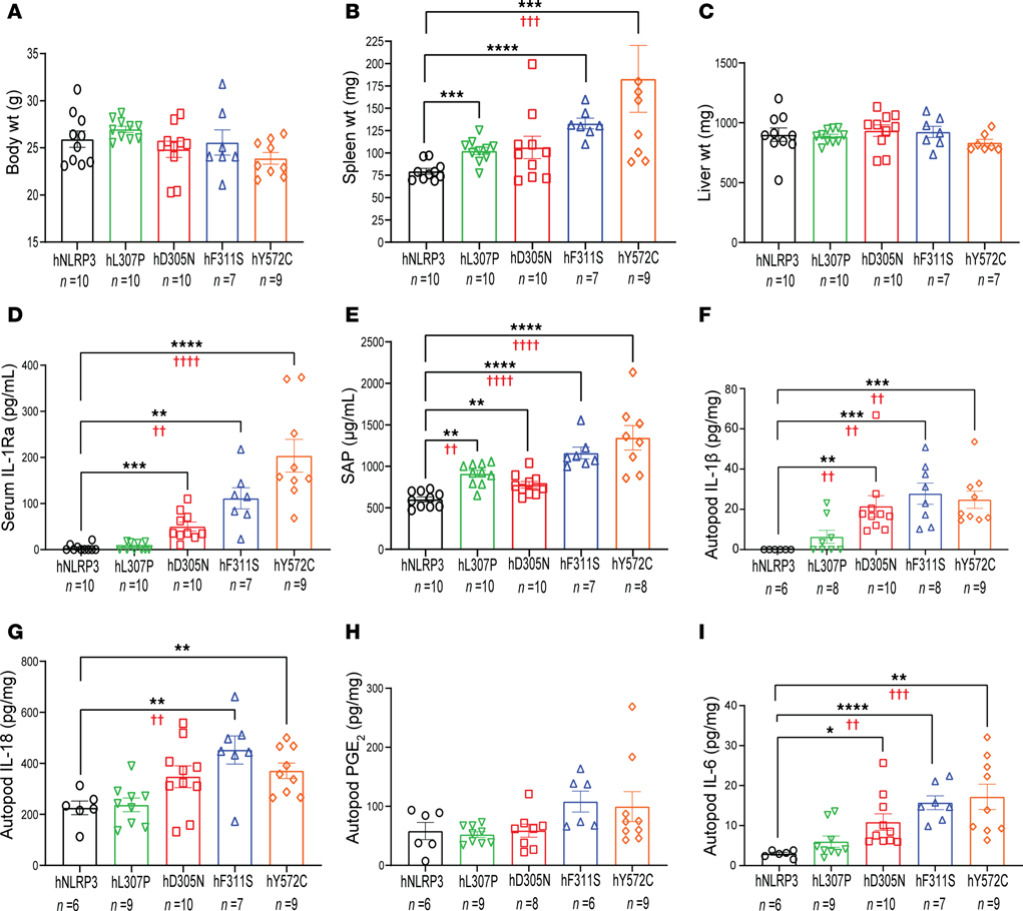
Phenotypic evaluation of mouse lines with a single copy of the common *NLRP3* allele or disease-associated variants. (**A**–**I**) Mouse lines were established from ES cell lines carrying a single copy of a CAPS-associated mutation. Female mice were cohoused at weaning, with each cage typically containing at least 1 mouse of each CAPS genotype as well as a female carrying the common *NLRP3* allele. At 9–10 months of age, the burden of autoinflammatory disease was evaluated through assessment of body weight (**A**), spleen weight (**B**), and liver weight (**C**). Serum levels of IL-1Ra (**D**) and SAP (**E**) were quantified via ELISA. For the analysis of arthritis-like disease development, ELISA was used to measure IL-1β (**F**), IL-18 (**G**), prostaglandin E_2_ (PGE_2_) (**H**), and IL-6 (**I**) levels in tissue homogenates from the autopods of NLRP3-mutant mouse lines. The number of samples (*n*) for groups is indicated below the genotype in each panel. Bars represent the mean ± SEM. Asterisks indicate significance for comparison of each CAPS line with the hNLRP3 cohort by 2-tailed unpaired *t* test (*P < 0.05, **P < 0.01, ***P < 0.001, ****P < 0.0001). Red daggers indicate *P* values from identical comparisons analyzed by 1-way ANOVA followed by Dunnett’s post-test. Linear trend in phenotype or outcome was assessed for phenotypes with 1-way ANOVA *P* < 0.05 by post hoc test for linear trend (GraphPad Prism): **B** (spleen weight), *P* = 0.0001; **D** (IL-1Ra), *P* = 0.0001; **E** (SAP), *P* = 0.001; **F** (IL-1β), *P* = 0.0001; **G** (IL-18), *P* = 0.0004; **I** (IL-6), *P* = 0.0001. Additional statistical evaluation of variation between individual lines is provided in Supplemental Table 2.

Mice heterozygous for the various CAPS mutations showed little difference in adult weight, though those carrying the Y527C mutation tended to be smaller (Figure 1A). On necropsy, a significant increase in spleen weight of the L307P, F311S, and Y572C lines was observed compared with that of the hNLRP3 line (Figure 1B). The weight of D305N spleens also increased. However, this increase did not achieve significance, likely because of the variation in this parameter between mice (Figure 1B). No increase in liver weight was observed in any of the lines (Figure 1C). IL-1Ra (IL-1 receptor antagonist) serves as the endogenous natural antagonist of IL-1β. Serum IL-1Ra levels are reported to be higher in CAPS than in healthy individuals. This increase likely reflects a homeostatic response initiated to limit damage due to pathological levels of IL-1β (40, 41). We found that the level of plasma IL-1Ra was significantly increased in D305N, F311S, and Y572C mice compared with mice carrying the L307P FCAS mutation or hNLRP3 allele (Figure 1D).

In patients with CAPS, increases in the levels of acute-phase reactants (APRs), such as C-reactive protein, have been reported (42–44). In mice, serum amyloid protein (SAP) is an APR that is strongly induced by a broad spectrum of inflammatory stimuli (45). We therefore examined the levels of SAP in the plasma of the 4 mutant mouse lines (Figure 1E). Plasma SAP levels in all CAPS lines were higher than those measured in hNLRP3 mice. The increase in levels generally paralleled the severity of the disease in patients with CAPS carrying the corresponding mutation, with the highest levels observed in the Y572C mice. Interestingly, the plasma SAP level measured in the L307P FCAS line was higher than that measured in D305N mice, suggesting the possibility of differences in the extent to which a given mutation activates distinct manifestations of the autoinflammatory disease phenotype.

### Development of arthropathy in mice carrying a CAPS-associated allele.

We previously reported that arthritic changes in the limbs of mice carrying the D305N mutation became progressively more noticeable with age (39). Furthermore, these changes corresponded to elevated levels of cytokines measurable in tissue homogenates from the affected joints. We therefore assessed how the genotype of the CAPS mice impacted the inflammation of the autopods of the 5 different mouse lines. To this end, tissue homogenates were prepared from the autopods of mice of each genotype, and cytokine and PGE_2_ levels were determined. The ELISA measuring IL-1β is expected to detect both mature and pro–IL-1β. IL-1β levels were below the level of detection in homogenates prepared from the hNLRP3 mouse line. In contrast, measurable levels of IL-1β were detected in tissue homogenates from all 4 mutant mouse lines (Figure 1F). Levels of IL-1β were lowest in mice carrying the mild L307P FCAS-associated mutation, and, although increases were seen in individual animals, overall levels were not significantly higher than in the hNLRP3 animals. The highest levels of IL-1β were measured in the homogenates prepared from the mice with high-impact mutations, D305, F311S, and Y572C.

High levels of IL-18 were observed in the autopod lysates prepared from hNLRP3 mice. Given the robust baseline presence of this cytokine, it is perhaps not surprising that no further increase was observed in samples from mice carrying a single L307P or D305N allele. In contrast, IL-18 was increased in lysates from mice carrying either of the other 2 high-impact alleles, F311S and Y572C (Figure 1G). PGE_2_ levels in the heterozygous L307P and D305N mice were similar to those measured in the hNLRP3 animals, while a small increase was observed in the PGE_2_ levels measured in Y572C and F311S homogenates (Figure 1H). IL-6 levels were increased in the tissue homogenates from all 4 mutant mice, with levels paralleling the reported relative clinical severity of these mutations (Figure 1I).

### LPS-mediated hypothermia and inflammation in the mice carrying a CAPS-associated allele.

**Figure 2:**
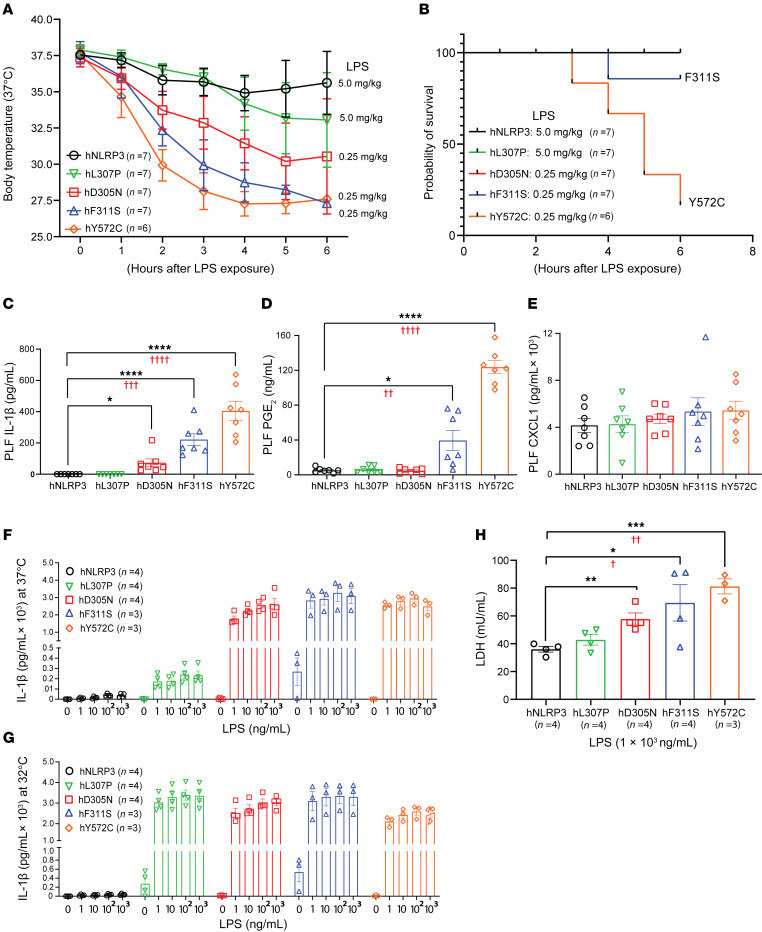
Response of the peritoneum and peritoneal macrophages in mice heterozygous for various CAPS alleles to LPS exposure. Mice heterozygous for the NLRP3 variants Y572C, F311S, and D305N received 0.25 mg/kg LPS i.p., whereas hNLRP3 and L307P mice received 5.0 mg/kg LPS. (**A**) Core body temperature was monitored for 6 hours postexposure. (**B**) Survival probability was assessed based on behavior and appearance, with genotype blinded. (**C**–**E**) In a parallel experiment, mice received 2.5 mg/kg LPS (i.p.); peritoneal lavage fluids (PLF) collected after 2 hours were analyzed for IL-1β (**C**), PGE_2_ (**D**), and CXCL1/KC (**E**) by ELISA. For **C**–**E**, *n* = 7 per genotype. (**F** and **G**) Peritoneal macrophages from the indicated genotypes were exposed to increasing LPS concentrations at either 37°C (**F**) or 32°C (**G**), and IL-1β release was measured after 4 hours. (**H**) Lactate dehydrogenase (LDH) release was measured in similarly prepared macrophages treated with 1 μg/mL LPS for 24 hours. For **A**, 1-way ANOVA with linear-trend testing indicated a genotype–phenotype relationship in the area under the core-temperature curve: *P* < 0.001. (**B**) Kaplan-Meier curves show survival probability after LPS exposure. Overall differences were confirmed by log-rank (Mantel-Cox) *P* < 0.05, with a monotonic trend supported by the log-rank test for trend *P* < 0.01. The Gehan-Breslow-Wilcoxon test *P ≤ 0.05 yielded a less significant result, indicating that group separation occurred mainly at later time points. (**C**–**E** and **H**) Asterisks indicate significance versus the hNLRP3 cohort by 2-tailed unpaired *t* test (*P < 0.05, **P < 0.01, ***P < 0.001, ****P < 0.0001). Red daggers indicate *P* values from identical comparisons by 1-way ANOVA with Dunnett’s post-test. Linear-trend testing yielded *P* < 0.0001 (**C** and **D**) and *P* < 0.0002 (**H**). Additional analyses are shown in Supplemental Table 3.

Monocyte cell death (pyroptosis) is a characteristic of CAPS (46). We previously reported that mice carrying the D305N mutation respond to peritoneal exposure to low doses of LPS with rapid hypothermia and the release of IL-1β by peritoneal myeloid cells (39). In contrast, mice carrying hNLRP3 are largely resistant to this level of endotoxin. Cohorts of mice carrying a single copy of the D305N, F311S, or Y572C allele were exposed to 0.25 mg/kg of LPS i.p. Based on the limited IL-1β release observed on analysis of ES cells carrying the FCAS-associated L307P mutation, we increased the LPS exposure to 5.0 mg/kg for this mouse line and for the control hNLRP3 animals. After endotoxin exposure, changes in core body temperature were monitored hourly (Figure 2A). Mice showing dramatic changes in well-being were removed from the experiment throughout by an observer while genotype was blinded. These animals were used to generate the “survival” curve shown in Figure 2B. Hypothermia in the D305N mice mimicked that previously reported, with a gradual drop in temperature that plateaued as animals began to recover from this inflammatory stimulus. A small but measurable change in core body temperature was observed in the L307P mice compared with the hNLRP3 animals that received the same dose of endotoxin (5.0 mg/kg). However, even with this increase in exposure level, the magnitude of the change was less than that in the D305N mice exposed to 20-fold less LPS. In comparison, the F311S and the Y572C mouse lines responded to endotoxin with both a decrease in the lag time before a change in temperature was observed and an increase in the magnitude of the temperature change. While differences were observed in the response of both lines compared with the D305N line, the changes were greater for the Y572C mice. Moreover, the increased endotoxin sensitivity of the Y572C line compared with the F311S line was readily apparent when comparing the number of mice of each genotype that required removal from the experiment for humane reasons (Figure 2B).

In a parallel experiment, we collected peritoneal exudates from mice 2 hours after endotoxin exposure to determine whether IL-1β levels could further distinguish the physiological impact of the gain-of-function mutations. The shorter duration allowed all mice to be treated with 2.5 mg/kg LPS delivered i.p. (Figure 2C). No increase in IL-1β was detected in peritoneal fluid from mice carrying hNLRP3 or from mice heterozygous for the FCAS mutation L307P. Consistent with our previous study, a significant increase was observed in the D305N line (39), with still higher levels in samples from F311S mice. In keeping with the comparison of hypothermic shock and body condition score after LPS exposure, IL-1β levels in Y572C mice exceeded those observed in all other lines. Because the peritoneal exudates were centrifuged to remove cells prior to ELISA, the IL-1β measured here reflects mature cytokine released following pyroptosis. We also measured PGE_2_ in the peritoneal exudate (Figure 2D). An increase in this lipid mediator was detected only in the 2 NOMID lines, F311S and Y572C, with levels again highest in Y572C samples. In contrast, no differences were observed in other LPS-responsive inflammatory mediators, such as CXCL1 (KC), in the peritoneal exudate (Figure 2E).

We next asked whether this difference in the release of IL-1β upon peritoneal exposure to LPS aligned with endotoxin-mediated release of IL-1β by peritoneal macrophages collected from the CAPS mouse lines. Peritoneal cells were collected from hNLRP3 mice and mice heterozygous for each of the 4 CAPS-associated mutations. Populations were enriched for macrophages by adherence to plastic and then exposed to medium alone or medium with LPS levels ranging from 1 to 1,000 ng/mL at 37°C (Figure 2F) and 32°C (Figure 2G). As expected, only low levels of IL-1β were detected in supernatants from macrophages from the hNLRP3 mice, even at the highest exposure levels. A concentration-dependent increase in IL-1β levels was measured in the medium collected from the D350N mice, plateauing at 100 ng/mL. In contrast, even at the lowest concentration, both F311S and Y527C showed maximal release. Two of the 3 primary cultures established from F311S mice showed very low levels of spontaneous release of IL-1β (Figure 2, F and G). While cells collected from F311S and Y572C are clearly more sensitive to endotoxin than cells from the D305N mice, the 2 NOMID/CINCA-associated mutations could not be ranked in this assay.

Consistent with the classification of L307P as an FCAS mutation, peritoneal cells isolated from the L307P mice released only low levels of IL-1β when cultured at 37°C and exposed to LPS (Figure 2F), and interestingly this release did not increase in response to increasing LPS levels. In contrast, when this experiment was carried out at 32°C, measurable basal release was observed (Figure 2G). A robust release of IL-1β, similar in magnitude to that of the CINCA/NOMID cell cultures, was measured in medium from the L307P cells after exposure even to the lowest concentration of LPS at 32°C. In comparing the response at 32°C and 37°C, a log increase in IL-1β release was observed in the L307P cells. In contrast, temperature had little impact on cytokine release by D305N, F311S, or Y527P peritoneal macrophages.

Using peritoneal macrophages collected from the 5 mouse lines, we also assessed LPS-triggered cell death by measuring lactate dehydrogenase (LDH) levels in the culture medium (Figure 2H). Although LDH is not specific for pyroptosis, it is widely used as a surrogate marker for NLRP3-mediated cell death (47, 48). As expected, given that this assay was carried out at 37°C, LDH levels in L307P cultures did not differ from control levels. However, LDH release increased according to genotype in the remaining 3 CAPS lines, and the linear-trend test was highly significant, supporting a strong genotype–phenotype relationship among the CAPS alleles.

### Sensitivity of CAPS-associated LPS-mediated IL-1β release to the NLRP3 activation inhibitor CP-456,773.

**Figure 3:**
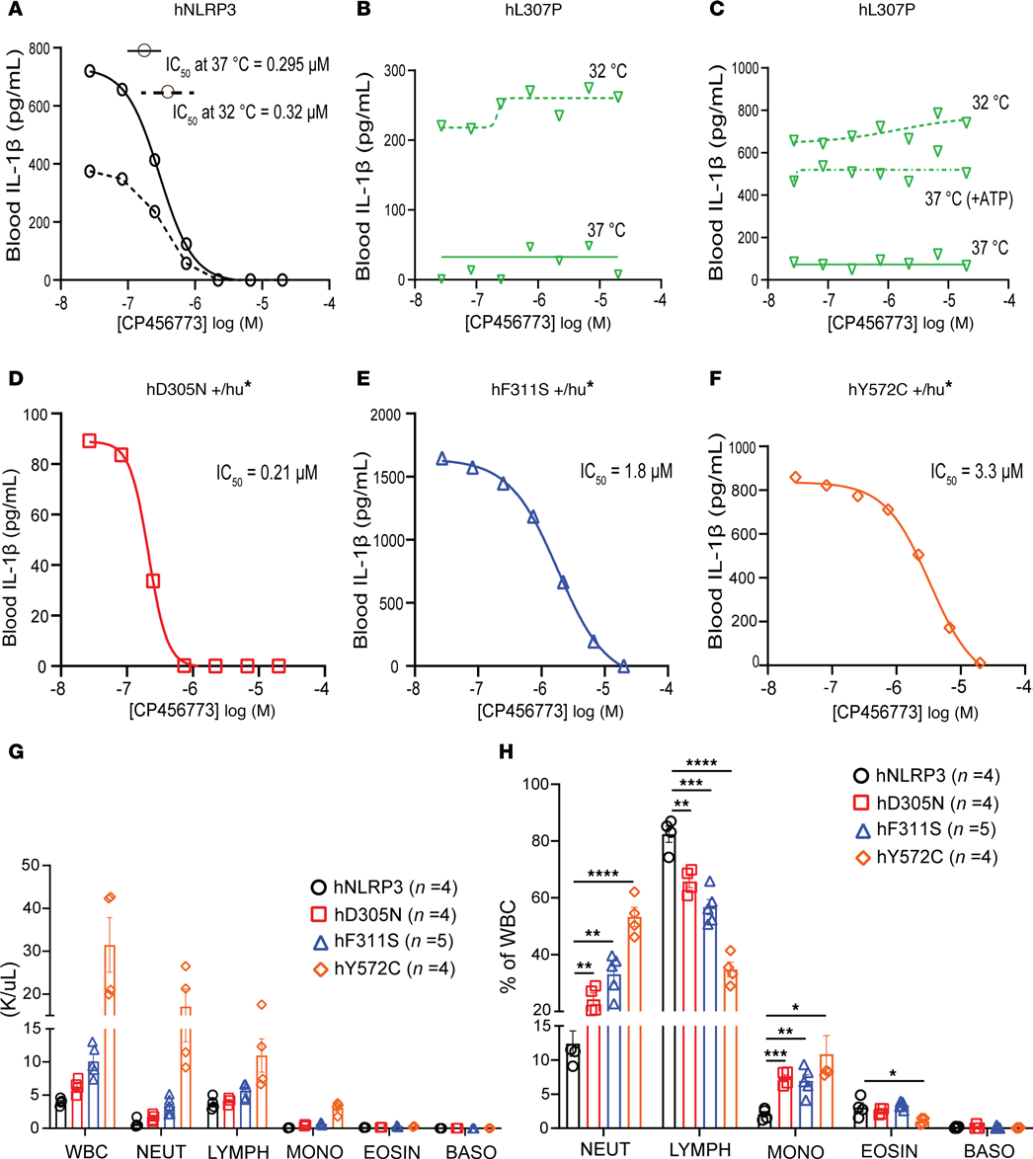
IL-1β release from blood of NLRP3-mutant mice and sensitivity to CP-456,773. Blood was collected from mice of the indicated genotype and exposed to LPS or LPS followed by ATP. In some experiments, blood was incubated throughout at 32°C rather than the standard 37°C. (**A**) Blood from mice homozygous for the common *NLRP3* allele (hNLRP3) released IL-1β after exposure to LPS and ATP. CP-456,773 inhibited release in a concentration-dependent manner. While release decreased when the assay was performed at 32°C, the IC_50_ was temperature independent. (**B**) No IL-1β release after LPS exposure of blood from mice homozygous for the L307P allele was observed at 37°C (solid line). In contrast, IL-1β release occurred at 32°C (dashed line), but this was not inhibited by CP-456,773. (**C**) IL-1β release from L307P blood was observed at 37°C when LPS exposure was followed by ATP (dashed/dotted line). This LPS/ATP-mediated release was insensitive to CP-456,773. (**D**–**F**) LPS exposure of blood from mice heterozygous for the D305N (**D**), F311S (**E**), and Y527C (**F**) alleles resulted in measurable IL-1β release. Release by all 3 mutants was inhibited in a concentration-dependent manner by CP-456,773. (**G** and **H**) Total white blood cell count (WBC) and leukocyte subpopulations in blood collected from the indicated number of hNLRP3, D305N, F311S, and Y572C mice were determined by CBC. (**G**) Thousands of cells per cubic milliliter (K/μL), with statistical evaluation detailed in Supplemental Table 4. (**H**) Leukocyte subpopulations as a percentage of total WBC, with differences between CAPS mice evaluated by 2-tailed unpaired *t* test (*P < 0.05, **P < 0.01, ***P < 0.001, ****P < 0.0001).

CP-456,773 (MCC950) was first identified as a potent inhibitor of IL-1β release from blood leukocytes and macrophages (47) and was later shown to mediate this effect by inhibiting NLRP3-dependent cytokine maturation (48). Using a method established to assess CP-456,773’s activity toward NLRP3 in whole blood (49), we tested its ability to inhibit IL-1β release from blood collected from hNLRP3 mice and mice heterozygous for D305N, F311S, and Y572C mutations (Figure 3, A–F). Anticipating a temperature-sensitive response, blood from L307P homozygous mice, rather than heterozygous animals, was included. This allowed comparison of CP-456,773’s inhibition of IL-1β release following exposure to LPS at 32°C alone as well as inhibition following canonical activation by LPS and ATP.

We first verified this assay using whole blood from hNLRP3 mice and assessed the impact of incubation temperature on the potency (expressed as IC_50_ value) of CP-456,773. Blood pooled from multiple hNLRP3 mice was incubated at either 32°C or 37°C with LPS. After 3 hours, samples were exposed to various concentrations of CP-456,773, and inflammasome assembly and IL-1β release were triggered by ATP. Not surprisingly, total IL-1β output was greater at 37°C than at 32°C. As expected, CP-456,773 inhibited IL-1β release in a temperature-independent manner, with an IC_50_ of approximately 0.3 μM (Figure 3A). To test the activity of CP-456,773 on mutant NLRP3, cells were treated with the compound prior to stimulation with LPS, as previous studies have shown that output of IL-1β from CAPS patient blood requires only LPS stimulation (50).

In blood from L307P mice cultured at 37°C, IL-1β release was minimal. In contrast, at 32°C, similar to results with peritoneal macrophages, IL-1β release increased 10-fold. However, release of IL-1β from LPS-treated L307P blood was not inhibited by even the highest CP-456,773 concentrations (Figure 3B). To determine if the failure of CP-456,773 to reduce IL-1β release was specific to cold-activated L307P inflammasomes, we examined IL-1β release from L307P blood cells after LPS priming and ATP exposure at 37°C. Despite using a different, presumably mutant-insensitive triggering mechanism, inflammasome assembly and IL-1β release remained resistant to CP-456,773 inhibition (Figure 3C). Similar sensitivity to cold temperature and insensitivity of the L307P inflammasome to CP-456,773 inhibition has been noted in an extrinsic system (21).

In contrast with IL-1β release triggered by the L307P inflammasome, release after LPS exposure from blood of mice carrying the MWS and NOMID/CINCA mutations was sensitive to CP-456,773 inhibition (Figure 3, D–F). While complete inhibition of IL-1β output was achieved, IC_50_ values for CP-456,773 showed variance between the different gain-of-function lines; values of 0.21 μM, 1.8 μM, and 3.3 μM were observed for D305N, F311S, and Y572C, respectively. We also verified that CP-456,773 was similarly effective in inhibiting IL-1β release in blood collected from mice homozygous for the CAPS-associated mutations. We tested this using blood from mice homozygous for the D305N allele. Based on 4 independent experiments, an IC_50_ of 0.79 ± 0.10 μM was determined (Supplemental Figure 2). In contrast with the FCAS mutation, and unlike reports where these mutations have been cold sensitive in extrinsic systems (21, 51), a temperature drop did not promote release of IL-1β from D305N, F311S, or Y572C blood isolates.

One possible explanation for the differences in IC_50_ between CAPS lines is that these differences reflect variation in the leukocyte composition of blood from the CAPS lines. To explore this possibility, a complete blood count (CBC) was performed on samples collected from the 3 mutant mouse lines and control hNLRP3 mice (Figure 3, G and H). Leukocytosis was evident in samples from our 4 mutant lines and was driven primarily by an increase in neutrophil numbers; both the absolute number of neutrophils as well as their percentage of total cell count were elevated relative to values observed in hNLRP3 blood. Both parameters paralleled disease severity, with extremely high levels observed in Y572C blood. However, differences in CBC did not parallel the ability of CP-456,773 to inhibit IL-β release, making it more likely that the structural difference between CAPS mutations alters drug sensitivity.

### Developmental phenotype of mice homozygous for the CAPS-associated mutations.

We previously reported that intercrossing mice heterozygous for the humanized *NLRP3* locus carrying the D305N mutation generated litters in which pups homozygous for the CAPS mutation were present at the expected frequency (39). The homozygous D305N/D305N pups could not be distinguished from their littermates at weaning, as the arthropathy characteristic of this line became apparent only with increasing age. The fertility of the D305N/D305N males did not differ from that of age-matched hNLRP3 mice. However, in contrast, while females could bear and nurture litters, their fecundity was variable and generally lower than that of hNLRP3 females in our vivarium, with many females bearing only 1 to 4 litters. Intercrossing mice heterozygous for the FCAS L307P mutation produced homozygous mice at expected frequencies, and both female and male homozygous mice were fertile. These mice could not be identified based on phenotype either at weaning or during early adulthood. While mice homozygous for the F311S mutation were also present in litters at the expected frequency, examination of the hind autopods of the pups at weaning identified the majority of F311S homozygous mice to the trained observer. Similar to the D305N and L307P mice, the arthropathy phenotypic of F311S/F311S mice became increasingly apparent with age. However, both male and female mice homozygous for the F311S CAPS mutation failed to reproduce when test-bred with hNLRP3 mice, necessitating the generation of homozygous F311S mice by intercrossing heterozygous animals. In contrast with the L307P, D305N, and F311S mouse lines, intercrossing mice heterozygous for Y527C did not yield homozygous pups at the expected numbers at weaning. Those still present in the litters at weaning were identifiable based on their small size. Examination of litters at earlier time points indicated that many of the Y527C pups were severely affected by the mutation and required removal from litters in the perinatal period. The number of homozygous Y572C pups present at early time points was variable, but increased numbers were observed in litters born to experienced dams. In summary, the overall health and survival observed in these 4 lines is consistent with the disease severity observed for the corresponding mutation in humans (Supplemental Table 1).

### Evaluation of phenotype in mice homozygous for the CAPS mutations.

Although patients with CAPS are heterozygous, we also examined homozygous animals not to model patient copy number but to amplify allele-specific differences and thereby better define the genetic relationships among CAPS variants. For these studies, mice homozygous for the L307P, F311S, or Y572C mutation were evaluated in the same experiments as mice homozygous for both D305N and hNLRP3. The D305N line was included in every phenotypic assessment because analyses of the heterozygous animals demonstrated that D305N exhibits an intermediate disease severity between the mild FCAS mutation and the severe NOMID/CINCA mutations. In addition, the D305N homozygous phenotype has been well characterized in prior publications and therefore provides a defined benchmark. Importantly, all comparisons were performed using cohoused hNLRP3 and D305N controls within each experiment, ensuring that no mutant line was ever evaluated in isolation and that every phenotype was interpreted relative to these 2 reference genotypes.

**Figure 4:**
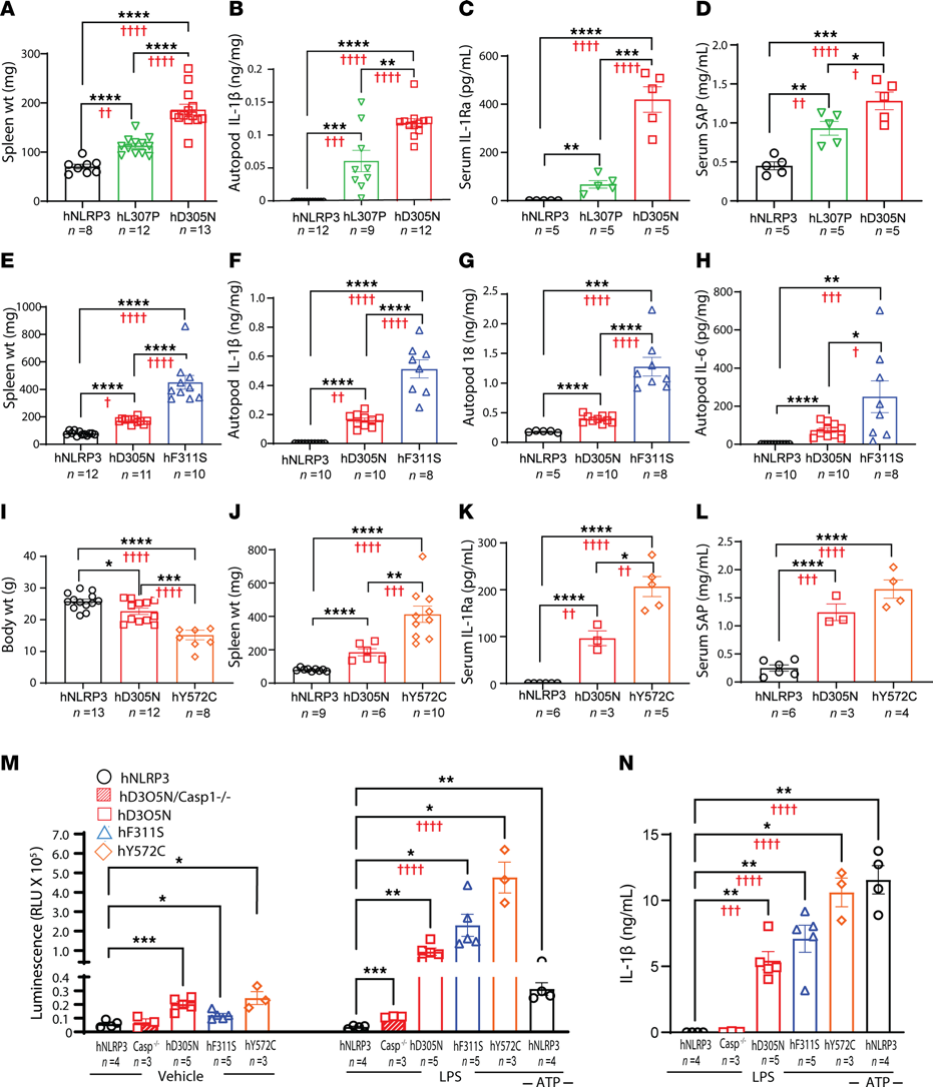
Comparison of inflammation in CAPS mice and primary macrophages homozygous for each mutant allele. (**A**–**L**) Tissues and fluids were collected at necropsy from mice of the indicated genotype. (**A**) Spleen weights in D305N and hNLRP3 mice compared with L307P mice. (**B**) IL-1β levels in autopod homogenates from hNLRP3, D305N, and L307P mice. (**C** and **D**) Serum IL-1Ra (**C**) and SAP (**D**) measured in hNLRP3, L307P, and D305N mice. (**E**) Spleen weight of hNLRP3, D305N, and F311S mice. (**F**–**H**) IL-1β (**F**), IL-18 (**G**), and IL-6 (**H**) levels in autopod lysates from hNLRP3, D305N, and F311S mice. (**I** and **J**) Body weight (**I**) and spleen weight (**J**) of hNLRP3, D305N, and Y572C mice at 8–12 weeks. (**K** and **L**) Serum IL-1Ra (**K**) and SAP (**L**) in hNLRP3, D305N, and Y572C mice. (**M** and **N**) Peritoneal macrophages were collected from mice of the indicated genotype to assess inflammasome responses to LPS. (**M**) Caspase-1 enzymatic activity in supernatant was quantified using a luminescence assay that reports relative light units (RLU) following 2 hours of LPS stimulation. (**N**) IL-1β levels in the same culture supernatants were measured by ELISA. hNLRP3 peritoneal macrophages stimulated with LPS and ATP served as positive controls. Vehicle-treated peritoneal macrophages from mice of each CAPS genotype and LPS-exposed macrophages from D305N Casp1/11-null mice, and from hNLRP3 mice served as specificity controls. Sample size (*n*) is indicated in each panel. Bars represent mean ± SEM. Asterisks indicate significance for comparisons among each CAPS line and hNLRP3 and between CAPS genotypes as determined by 2-tailed unpaired *t* test (*P < 0.05, **P < 0.01, ***P < 0.001, ****P < 0.0001), with additional comparisons in Supplemental Table 5. Red daggers denote *P* values derived from identical comparisons assessed by 1-way ANOVA with Tukey-Kramer post-test (**A**–**L**), 2-way ANOVA (**M**), or 1-way ANOVA (**N**), with Dunnett’s post-test.

Mice heterozygous for the FCAS L307P mutation showed only modest increases in inflammatory parameters compared with hNLRP3 mice, with significant differences limited to spleen weight and serum SAP levels (Figure 1, B and E). These 2 phenotypes were also significantly increased in homozygous L307P mice relative to hNLRP3 animals (Figure 4, A and D). Total IL-1β levels in autopod homogenates and serum IL-1Ra levels were more clearly elevated in the homozygous L307P mice and reached statistical significance (Figure 4, B and C), whereas these analytes did not differ significantly in L307P heterozygotes (Figure 1, D and F).

Comparison of inflammatory parameters between homozygous D305N and L307P mice showed that the L307P line, consistent with individuals with FCAS, exhibited a markedly milder phenotype than mice carrying MWS/NOMID-associated variants (Figure 4, A–D). For all parameters examined, inflammation was greater in homozygous D305N than in L307P mice.

We compared homozygous D305N, F311S, and hNLRP3 cage mates to verify the parameters that differed between these mutations in heterozygous animals. The relative intensity of inflammation between the 2 homozygous lines indicates that, consistent with the limited case reports of patients with F311S mutations, this variant is likely to produce a more severe phenotype than D305N. Spleen weights were nearly 2-fold higher in F311S mice compared with D305N animals (Figure 4E). Likewise, levels of IL-1β, IL-18, and IL-6 in autopod lysates from F311S mice were approximately 2-fold higher than in D305N mice (Figure 4, F–H). All 4 inflammatory parameters were significantly elevated in the homozygous F311S line compared with the D305N line.

As noted above, a subset of homozygous Y572C mice survived the perinatal period and continued to gain weight until 8–12 weeks of age, allowing comparison with age-matched homozygous D305N animals and hNLRP3 controls. As expected, Y572C weighed significantly less than the D305N controls (Figure 4I). All 4 CAPS lines showed some increase in spleen size; however, the enlargement in Y572C homozygous mice was striking, with spleens reaching approximately 4 times the size observed in healthy animals (Figure 4J). Serum levels of SAP and IL-1Ra were also substantially higher in Y572C mice than in both hNLRP3 and D305N animals, consistent with findings in the heterozygous mice (Figure 4, K and L). Across nearly all parameters measured, the inflammatory disease conferred by the Y572C mutation was markedly more severe than that observed in the D305N line.

### Evaluation of caspase-1 activity as a measure of inflammasome function.

We collected peritoneal macrophages from 8-week-old hNLRP3 mice and from mice homozygous for each of the CAPS alleles to compare inflammasome function, assessed by caspase-1 activity in the culture medium in response to LPS exposure (Figure 4M). Macrophages from D305N Casp1/11^−/−^ mice were included as a control for nonspecific substrate cleavage. Vehicle treatment for 2 hours produced a small increase in spontaneous caspase-1 signal in medium collected from the CAPS cultures, but this signal did not correlate with mutation severity. In contrast, LPS exposure produced a robust increase in caspase-1 activity that aligned with mutation severity. Caspase-1 activity in response to LPS showed the same mutation-dependent trend as the IL-1β release from these same cultures (Figure 4N).

### CNS autoinflammation in the CAPS mouse lines.

**Figure 5:**
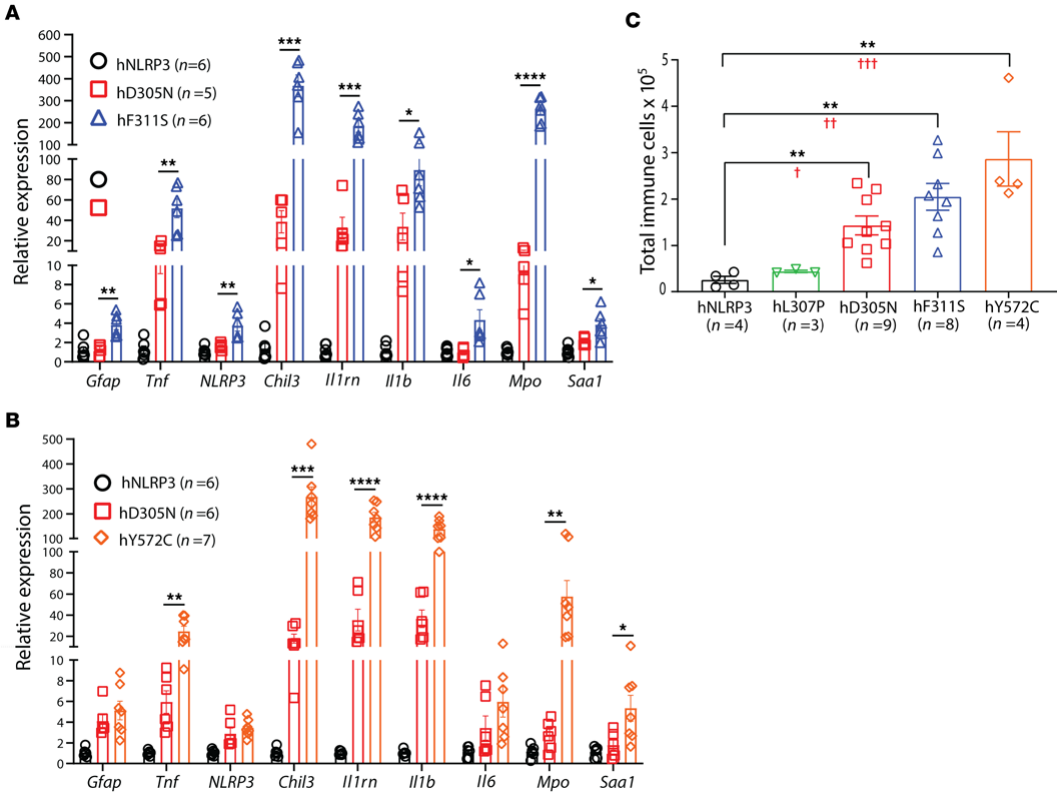
Autoinflammatory disease in the CNS of CAPS mouse lines. (**A** and **B**) CNS inflammation was assessed by quantifying mRNA expression of inflammatory mediators and *Gfap*, an established marker of astrogliosis. ΔΔCt values for hNLRP3 mice were set to 1; mutants are shown relative to this. (**A**) Transcript abundance was measured in whole-brain tissue collected from 7-month-old hNLRP3, D305N, and F311S mice. (**B**) The same panel of inflammation-related transcripts was evaluated in whole-brain mRNA prepared from 8- to 12-week hNLRP3, D305N, and Y572C mice. Analytes include *Il1b* (IL-1β), *Chil3* (chitinase-like 3), *Il1rn* (IL-1Ra), *Tnf* (tumor necrosis factor), *Gfap* (glial fibrillary acidic protein), *Mpo* (myeloperoxidase), *Il6* (interleukin-6), *Saa1* (serum amyloid A1), and *NLRP3*. These markers reflect inflammasome-driven cytokine activity, myeloid activation, and astrocytic reactivity characteristic of CAPS pathology. (**C**) CNS immune cell infiltration was quantified by flow cytometry. Leukocyte-enriched fractions were prepared from whole-brain homogenates by density gradient separation, and immune cells were enumerated based on CD45 expression. Sample sizes (*n*) for each genotype are indicated in each panel. Data are presented as mean ± SEM. For **A** and **B**, statistical comparisons were performed by 2-tailed unpaired *t* test between hD305N and hF311S (**A**) and between hD305N and hY572C (**B**). For **C**, asterisks indicate significance for comparisons of each CAPS line to hNLRP3 by 2-tailed unpaired *t* test (*P < 0.05, **P < 0.01, ***P < 0.001, ****P < 0.0001). (**C**) Red daggers indicate *P* values for the same comparisons evaluated by 1-way ANOVA with Dunnett’s post-test. (**C**) A linear-trend analysis was performed using 1-way ANOVA followed by the GraphPad Prism post hoc test for linear trend, revealing a significant monotonic relationship (*P* = 0.0001). Pairwise comparison between individual CAPS lines is provided in Supplemental Table 6.

CNS involvement is observed in patients at the more severe end of the CAPS clinical continuum (MWS/NOMID/CINCA) (52, 53). Symptoms range from headaches, eye inflammation, and hearing loss to severe meningitis with hydrocephalic changes that affect CNS function and cranial development. We previously reported sterile meningitis in D305N mice and showed that brain inflammation increased mRNA levels of inflammation-related genes (54). We therefore collected the right hemisphere from saline-perfused control mice and CAPS mice and prepared mRNA for qPCR analysis. All CAPS mice in this analysis were homozygous for their respective alleles. Transcript levels were normalized to those observed in brains from hNLRP3 mice. Expression in 7-month-old D305N mice was compared with expression in age-matched F311S mice, and the same transcript panel was analyzed in 8- to 12-week-old Y572C mice relative to age-matched D305N animals. Expression of all transcripts examined was significantly higher in RNA from F311S brains than from D305N brains (Figure 5A). Analysis of the same transcript set in 8- to 12-week-old D305N and Y572C mice again showed a marked increase in inflammation-related gene expression in Y572C brains relative to age-matched D305N samples (Figure 5B).

To further quantify CNS involvement across lines, we measured total leukocytes in tissue homogenates prepared from the brain and leptomeninges. Leukocyte-enriched fractions were collected by Percoll gradient from homogenates of 2- to 5-month-old hNLRP3 and CAPS mice, and CD45^+^ cells were quantified by flow cytometry (Figure 5C). The total number of leukocytes recovered was genotype dependent: With the exception of L307P, all CAPS lines showed significantly higher cell numbers than hNLRP3 controls. Counts from D305N mice, although elevated relative to hNLRP3, were lower than those from F311S and Y572C mice. The highest leukocyte numbers were recovered from Y572C samples.

## Discussion

We report here a comparative analysis of mice carrying 4 distinct NLRP3 pathogenic variants that span the human CAPS severity spectrum. In these models, the mouse *Nlrp3* locus is replaced with the human gene encoding either the common allele or 1 of 4 CAPS-associated alleles. These lines extend our understanding of CAPS and demonstrate how NLRP3 activity drives inflammation across diverse tissue compartments. Notably, their phenotypes differ from those of earlier models in which human CAPS mutations were knocked into the mouse *Nlrp3* gene, highlighting limitations of traditional knockin approaches. Our findings suggest that, for some genes, species-specific expression patterns and/or intramolecular epistasis can limit the ability of knockin models to predict the functional consequences of human disease–associated missense variants.

Unlike the mouse lines in which FCAS mutations were introduced into the mouse NLRP3 protein, mice that carry and express the human FCAS protein under control of a human promoter recapitulate many aspects of the human syndrome. This includes the general good health of the mice, their longevity, and their excellent reproduction — even when homozygous for the FCAS mutation — despite measurable elevations in markers of systemic inflammation, including elevated SAP, increased IL-1β levels in joints, and a modest increase in spleen size, likely reflecting increased myelopoiesis. A defining feature of FCAS is the temperature sensitivity of the L307P inflammasome activity. This can be measured ex vivo as a temperature-dependent increase in IL-1β release from monocytes isolated from patients with FCAS (50). Our L307P line reproduced this phenotype, as LPS-treated primary peritoneal macrophages released IL-1β when cultured at 32°C compared with 37°C. In contrast, knockin FCAS models have been reported to develop severe autoimmune disease and often die early in life (27). Thus, the L307P line is poised to provide a robust model for further study of the pathophysiology of FCAS. For example, it will be interesting to determine the impact of environmental temperature changes, as well as lineage-specific expression of the mutant protein, on these disease parameters. At a more general level, the conservation of the temperature-sensitive characteristics of this mutation — demonstrated in primary macrophage populations and occurring alongside an overall mild systemic phenotype in our model — supports the central hypothesis of this study of CAPS lines: namely, that NLRP3 mutations are “buffered” by intramolecular protein interactions and that these interactions may be context sensitive and therefore species specific.

The phenotypes of the 3 mouse lines carrying NOMID/CINCA-associated mutations further underscore the importance of evaluating human missense mutations in the context of the human NLRP3 protein. D305N is a common variant in patients with MWS and NOMID/CINCA, and clinical reports indicate that CNS involvement is generally less pronounced in D305N carriers than in those with other NOMID/CINCA mutations, including Y572C. Several multigenerational cases of D305N suggest a comparatively milder phenotype, whereas most NOMID/CINCA mutations are de novo and, before anti–IL-1β therapy, rarely transmitted because few patients survived to adulthood (36). Y572C is one of the most frequent NOMID/CINCA mutations and is associated with severe disease, including failure to thrive and extensive CNS involvement leading to permanent neuronal damage. The F311S mutation has also been identified in several patients with NOMID/CINCA, and we included it here to test whether our model could resolve its pathogenicity across organ systems relative to D305N and Y572C. Notably, recent studies using an extrinsic reconstitution platform failed to distinguish these mutations based on ASC speck formation (21).

A possible explanation for the phenotypic differences between our CAPS lines and models in which CAPS mutations were knocked into the mouse gene is the species origin of the promoter driving mutant NLRP3 expression. This idea is supported by our recent work restoring the mouse promoter to both the hNLRP3 and hD305N loci in our humanized mice (54). These studies revealed species-specific promoter effects sufficient to modulate aspects of the D305N phenotype; notably, and surprisingly, CNS inflammation was reduced when the mouse promoter was reinstated. However, systemic inflammation — including all disease parameters reported here — was largely unchanged when the expression of the human *NLRP3* transcript carrying the D305N mutation was regulated by the mouse promoter. In contrast, a D305N-knockin model exhibited a far more severe phenotype, including perinatal lethality, impaired growth, joint inflammation, and death by weaning (28). Given the absence of severe systemic disease in promoter-restored humanized mice, promoter differences alone cannot account for the striking divergence between our model and the D305N-knockin line.

An alternative rationale for the difference in phenotype between models generated by knockin versus those generated by syntenic replacement is that it reflects species differences in the structure of the NLRP3 protein, as the amino acid substitution in our models is made in the context of the human rather than the murine version of the protein. Intramolecular epistatic interactions between amino acids located in different regions of the NLRP3 protein may modify the impact of substitution of a functionally critical residue, potentially making it more difficult to predict the severity of a mutation based on primary structure alone. Neutral amino acid substitutions that confer no selective benefit when they first arise may potentiate or support tolerance of subsequent function-altering mutations through direct or indirect structural mechanisms. Theoretical and empirical results indicate that such compensatory interactions can exert a strong influence on the trajectories of protein evolution (55). High levels of intramolecular epistasis may be particularly beneficial in molecules such as NLRP3, in which ability to alter primary structure in response to environmental challenges may increase the fitness of the organism. Because human and mouse NLRP3 have followed different evolutionary pathways, they may differ in their tolerance for a given CAPS mutation (55, 56), and thus divergence in epistatic interactions may account for differences observed between our models, which express human *NLRP3* *v*ariants, and those expressing knocked-in variants of the mouse protein. It also remains unclear how differences in the structure of the NLRP3 protein, both between species and between variant alleles, may impact their response/sensitivity to DAMPS and PAMPS, as the full array of these has not been identified.

With increasing structural insight into NLRP3, it may become feasible to predict the phenotypic impact of CAPS mutations in silico by defining intramolecular epistatic interactions that influence NLRP3 function. Cryo-EM data, computational models, and a crystal structure have helped define the spatial organization of NLRP3 domains and motifs, including the ADP/ATP binding pocket and key protein–protein interfaces. D305 lies within the Walker B motif of the nucleotide-binding domain, and computational analyses suggest a role in ATP binding, which promotes the structural transition required for monomer interactions and assembly of the NLRP3 inflammasome scaffold (57, 58). It has further been proposed that D305 coordinates with Mg²^+^ to facilitate ATP hydrolysis (59, 60). Although the NLRP3-NACHT crystal structure contained ATP during purification, ADP occupied the nucleotide-binding pocket (61). In this structure, the D305 side chain does not directly contact ADP, but ADP is thought to stabilize an inactive conformation. The D305N substitution has therefore been proposed to promote ADP release and replacement with ATP (61).

The roles of F311S and Y572C are less clear from existing structural and modeling studies. Computational analyses predict that F311S is neither directly involved in ADP/ATP binding nor positioned at a protein–protein interface (58), yet consistent with its association with the NOMID/CINCA spectrum, our in vivo studies show a severe phenotype in mice carrying this variant. Y572C is positioned differently depending on context: Modeling places it near the ATP-binding pocket (58), whereas the crystal structure does not identify it as a residue contributing to ADP binding (61). A fuller understanding of how individual CAPS mutations alter function will benefit from additional x-ray structures of mutant NLRP3 oligomers and refined modeling studies (59). Although effective and essential for evaluation of the expanding number of NLRP3 variants, extrinsic high-throughput screens failed to distinguish the 3 NOMID/CINCA mutations (21). As suggested previously (62), this may reflect mutation-specific effects across the diverse myeloid cell types expressing NLRP3 and contributing to CAPS pathogenesis.

From a pharmacological perspective, NLRP3 inhibition is an attractive therapeutic strategy for a broad range of inflammatory disorders (63), and phase I trials have already evaluated small-molecule inhibitors in CAPS (64). Prior in vitro studies using CAPS patient blood or PBMCs examined whether individual mutations remained sensitive to the prototype inhibitor CP-456,773/MCC950 (48, 65, 66). Most mutations associated with MWS and NOMID/CINCA, including D305N, retained CP-456,773 sensitivity (65), consistent with our report that IL-1β output from D305N mice is also inhibited by CP-456,773 (67). Other variants we evaluated — L307P, F311S, and Y572C — were not previously tested. Notably, several FCAS mutations (E527K, Y565I, Y565N) showed reduced sensitivity (65). Thus, FCAS mutations as a group appear less responsive to CP-456,773 (21). Structural studies indicate that CP-456,773 binds a pocket formed at the intersection of multiple NACHT subdomains, stabilizing an inactive NLRP3 conformation (61, 68, 69). Although adjacent to the ADP-binding site, the drug-binding pocket is distinct. The basis for reduced inhibitor sensitivity among FCAS mutations remains unresolved.

The influence of modifier loci was minimized in our study because all mice were co-isogenic 129 lines. Environmental variation was also reduced by cohousing mice of different genotypes at weaning, promoting shared microflora. Despite these controls, we still observed substantial variability in inflammatory severity among individual CAPS mice; for example, a subset of Y572C animals survived to adulthood. These findings suggest that stochastic factors contribute to disease progression. Future studies exposing CAPS mice to defined environmental stimuli may clarify gene–environment interactions that modulate autoinflammatory phenotypes, and congenic lines carrying the same mutant allele may help assess the role of modifier loci. Additionally, future studies will seek to better understand the mechanisms by which the various mutations affect translation and posttranslational activation of the NLRP3 gain-of-function alleles.

In summary, the range of disease severities observed across the 4 CAPS mouse lines demonstrates that the human clinical continuum — from FCAS to the most severe NOMID/CINCA mutations — can be recapitulated in this model organism. Our findings show that modeling the relative severity conferred by different NLRP3 mutations requires introducing these variants into the human, rather than the mouse, NLRP3 coding sequence, despite greater than 86% amino acid conservation. By inference, these results suggest that nonconserved residues exert critical epistatic effects on NLRP3 function.

## Methods

### Sex as a biological variable.

Previous experiments did not detect sex differences in the CAPS-like phenotype. Therefore, to allow cohousing of animals from different litters, females were used for most experiments.

### Reagents.

Details regarding materials, equipment, its application, and software used for the experiments and figure preparation are provided in Supplemental Table 7.

### Animal breeding and housing.

Mice were maintained in a pathogen-free facility in individually ventilated cages with irradiated bedding. Animals were fed ad libitum. Cages were enriched with Nestlets and a red “mouse house” that reflects and transmits mainly red light, which mice detect poorly, thereby reducing visual disturbance and stress. Drinking water was purified by reverse osmosis and supplied by an automated system. All mice were bred and reared under consistent conditions at University of North Carolina at Chapel Hill and were group-housed at weaning to ensure that mice with different *NLRP3* alleles were cohoused and exposed to the same microenvironment during aging.

### Generation of mice carrying a humanized NLRP3 locus.

Mice in which the 35 kb mouse *Nlrp3* locus was replaced with the common human *NLRP3* locus have been described previously (39). The common human *NLRP3* allele introduced (from BAC RP11-243D8, chr1:247,570,114–247,618,312) corresponds to the RefSeq transcript NM_004895.5 (protein NP_004886.3; UniProt Q96P20). The targeted locus was sequenced to confirm correct replacement of the mouse locus in the ES cells. Introduction of SNPs into this human segment to generate the D305N CAPS-associated allele has also been reported (39, 54). The same approach was used to generate mice carrying the L307P, F311S, or Y572C alleles. In all cases, the humanization event was confirmed by PCR, Southern blot, and sequence analysis. All mice are co-isogenic 129S6.

### Isolation and culture of peritoneal macrophages and LPS stimulation.

Peritoneal macrophages were collected by peritoneal lavage with 4 mL Hank’s buffer lacking Ca²^+^ and Mg²^+^. Cells were allowed to adhere to Petri dishes in macrophage culture medium (RPMI 1640, 10% FBS, 10 mM HEPES, 2 mM l-glutamine, 1× penicillin/streptomycin, 0.1 mM β-ME) at 37°C in a 5% CO_2_ incubator. After 2 hours, dishes were washed 3 times with culture medium to remove nonadherent cells. Macrophages were detached using cold PBS/10 mM EDTA, counted, and plated in 96-well, flat-bottom plates at 1×10^5^ cells/well. Cells were incubated overnight before stimulation. To induce cytokine release, macrophages were primed for 4 hours with the indicated LPS concentration in culture medium. Supernatants were collected at the end of the incubation period for cytokine analysis.

### LPS-induced IL-1β release in the peritoneum.

Mice received an i.p. injection of LPS in saline at the indicated dose. Core body temperature was monitored hourly with a rectal probe. Observers blinded to genotype assessed animals, and mice with a body condition score 2 were euthanized. In some experiments, peritoneal exudate was collected 2 hours after LPS exposure by lavage with 3 mL PBS. The recovered fluid, containing peritoneal cells and inflammatory mediators, was centrifuged at 500*g* to remove cells. Supernatants were analyzed for cytokines and lipid mediators.

### Quantification of cytokines and inflammatory mediators.

Autopod homogenates, including the tarsus, were prepared as described previously (70) with protease inhibitor tablets. Cytokine and PGE_2_ levels in homogenates, serum, peritoneal lavage fluid, and culture supernatants were measured using commercial ELISA kits according to the manufacturers’ instructions.

### LPS/ATP whole-blood assay.

Blood was collected from the left ventricle using a 26-gauge needle and transferred immediately to an Eppendorf tube containing 10 U heparin. Heparinized blood was diluted 1:0.5 in RPMI 1640 with 20 mM HEPES (pH 7.3). LPS (1 μg/mL) and CP-456,773 or vehicle were added to achieve the indicated final concentrations. Samples (190 μL/well) were transferred to a 96-well plate and incubated for 3 hours at the indicated temperature. In some samples, after the 3 hours of LPS priming, 10 μL of 100 mM ATP (5 mM final) was added and incubated for an additional 30 minutes. Plates were centrifuged at 500*g* at the end of the incubation, and plasma/medium supernatants were collected for cytokine analysis.

### LDH assay.

LDH release was measured in supernatants from peritoneal macrophages treated with 1 μg/mL LPS for 24 hours using the LDH Cytotoxicity Detection Kit according to the manufacturer’s instructions. An LDH standard curve was used to determine LDH concentration.

### Caspase-1 activity assay.

Caspase-1 activity in culture supernatants was measured by luminescence using the Caspase-Glo 1 Inflammasome Assay Kit following the manufacturer’s protocol. Peritoneal macrophages were seeded at 2 × 10^5^ cells/well in 96-well, tissue culture–treated plates in RPMI-1640 with 10% FBS, 10 mM HEPES, 0.1 mM β-ME, 2 mM l-glutamine, and 1× penicillin/streptomycin. The next day, cells were primed for 2 hours with 100 μL of 1 μg/mL LPS in RPMI 1640 containing 1% FBS and the additives listed above. For hNLRP3 samples, primed cells were then stimulated with 5 μL of 100 mM ATP (5 mM final) for 30 minutes. For caspase-1 activity measurement, 50 μL of supernatant was mixed with 50 μL Caspase-Glo reagent (Z-WEHD substrate + MG-132) in white, 96-well plates and incubated for 1 hour. Luminescence was recorded.

### Gene expression analysis.

RNA was isolated from tissues, and cDNA was synthesized with a cDNA Reverse Transcription Kit following the manufacturer’s instructions. Quantitative real-time PCR was performed using TaqMan Universal Master Mix with commercial probes and primers. Reactions were run in duplicate in 20 μL volumes with the following conditions: 50°C for 2 minutes, 95°C for 10 minutes, then 40 cycles of 95°C for 15 seconds and 60°C for 1 minute. Gene expression was normalized to 18S RNA using the ΔCT method, and relative expression was calculated using the ΔΔCT method. Expression in mutant samples was normalized to cohoused hNLRP3 controls.

### Isolation of brain leukocyte–enriched populations.

Immune cells were enriched from whole brain as described previously with modifications (71, 72). Perfused brains were diced and transferred to a MACS C tube containing 2 mL dissociation medium (RPMI 1640, 0.4 mg/mL Collagenase Type 4, 2% FBS, 2 mM HEPES, 50 μg/mL DNase I) and incubated for 20 minutes at 37°C with gentle inversion every 5 minutes. Digestion was stopped by adding 200 μL FBS. Samples were dissociated and filtered through a 40 μm strainer. The suspension was centrifuged at 250*g* for 4 minutes, and the pellet was resuspended in 10 mL 37% isotonic Percoll with 10% FBS in PBS. One milliliter PBS/10% FBS was gently overlaid, and gradients were centrifuged at 500*g* for 30 minutes at 4°C with no brake. Leukocyte-enriched populations were collected from the bottom fraction, washed in PBS, and used for flow cytometry.

### Flow cytometry.

Percoll-enriched immune populations (2 × 10^5^ cells) were analyzed as described previously (73). Cells were incubated with anti-CD16/32 in FACS buffer (PBS + 2% FBS) for 15 minutes at 4°C to block Fc receptors, then stained with fluorophore-conjugated antibodies against CD11b and CD45 for 40 minutes at 4°C in the dark. Cells were washed twice and resuspended in FACS buffer, and 150 μL of each sample was analyzed by flow cytometry. Compensation matrices were generated automatically using single-stain controls. Data were analyzed in FlowJo v10.6.1.

### Clinical chemistry.

Hematological analysis was performed by the University of North Carolina Animal Clinical Chemistry Core. Approximately 100 μL of unclotted, EDTA-treated blood was submitted per animal.

### Statistics.

Statistical methods and group sizes are indicated in each figure and corresponding legends. Analyses were performed using GraphPad Prism. *P* values less than 0.05 were considered statistically significant.

### Study approval.

At the University of North Carolina at Chapel Hill, all animal work complies with the NIH *Guide for the Care and Use of Laboratory Animals* (National Academies Press, 2011) and US Department of Agriculture guidelines and is approved by the Institutional Animal Care and Use Committee.

### Data availability.

Supporting data values for figures are available in the accompanying Supporting Data Values Excel file.

## Author contributions

JNS, CAG, IA, JPYT, and BHK conceived the study. JNS, MTN, and BHK designed and performed the experiments. JNS, MTN, and BHK created reagents. JNS, MTN, and BHK acquired and analyzed data. JNS, IA, CAG, and BHK drafted the manuscript. JNS, MTN, CAG, IA, JPYT, and BHK revised the manuscript. JNS and BHK supervised the study. JNS, MTN, CAG, IA, JPYT, and BHK discussed results and commented on the manuscript.

## Funding support

This work is the result of NIH funding, in whole or in part, and is subject to the NIH Public Access Policy. Through acceptance of this federal funding, the NIH has been given a right to make the work publicly available in PubMed Central.

- NIH/NIAID R01AI158314 to JP-YT and BHK.
- NIH/NIAID R21AI166471 to BHK.
- NIH/NIAMS AR061491 to BHK.
- NIH/NHGRI to BHK (UM1HG012003).
- NIH/NCI to Cytometry Core Facility (P30CA016086).

## Supplementary Material

Supplemental data

Supporting data values

## Version 1 03/09/2026

Electronic publication
