# Supplementary material for: Mice humanized by syntenic replacement with full-length NLRP3 disease-associated variants model the clinical cryopyrinopathy continuum
Source: JCI Insight. 2026 Mar 9;11(5):e194677. doi: 10.1172/jci.insight.194677 (PMC13041679; doi:10.1172/jci.insight.194677)
Supplement: Supplemental data [file jciinsight-11-194677-s053.pdf]

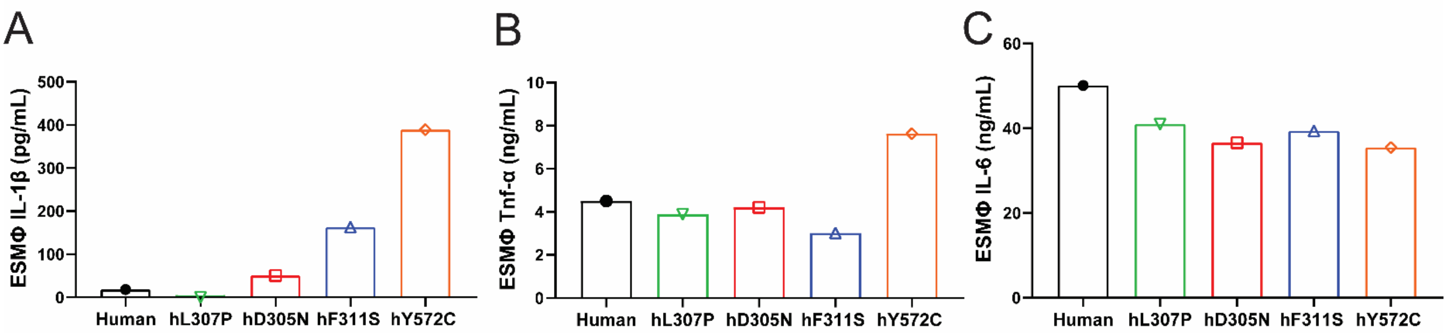

Supplemental Figure 1. Phenotypic evaluation of ES cell–derived macrophages humanized for the common *NLRP3* allele or disease-associated variants.

The endogenous mouse *Nlrp3* locus in an ES cell line was deleted and reconstituted with one of five DNA segments, each differing only in the human *NLRP3* allele they encode. These alleles include the common *NLRP3* variant and mutations identified in CAPS patients spanning a range of clinical severities. Equivalent numbers of ES cells of each genotype were differentiated into macrophage-like cells using a published protocol (1). Briefly, ES cells were cultured on 0.1% gelatin–treated dishes in DMEM supplemented with 15% FCS, 10 ng/mL LIF, and 1.5×10^{-4} M monothioglycerol (MTG). For embryoid body formation, cells were trypsinized and plated in bacterial-grade Petri dishes at 7.5×10^{3} cells/mL in IMDM containing 15% FCS, 2 mM glutamine, 300 µg/mL transferrin, 50 µg/mL ascorbic acid, 5% PFHM-II, and 4×10^{-4} M MTG. Embryoid bodies were harvested on day 6, washed once with IMDM + 10% FCS, and cultured at 1×10^{6} cells/mL in IMDM supplemented with 15% L929 conditioned medium (CM), 1 ng/mL IL-3, and 1.5×10^{-4} M MTG. After 48 hours, non-adherent macrophage precursors were collected and cultured in IMDM with 15% L929 CM, 10% FCS, and 1.5×10^{-4} M MTG. Adherent macrophages developed over the following 2–3 days and were used after day 7. Cytokine release following 24-hour LPS exposure was quantified. IL-1β release varied by *NLRP3* genotype in a pattern that reflects the clinical severity observed in humans (**A**). Cells expressing the L307P allele showed minimal IL-1β secretion, whereas F311S- and Y523C-expressing cells exhibited markedly elevated release, and D305N-expressing cells displayed intermediate levels. In contrast, TNF-α (**B**) and IL-6 (**C**) levels measured from the same supernatants did not correlate with disease severity. Although this was a single pilot experiment containing all five alleles, the striking allele-specific differences in IL-1β release provided an important proof-of-concept. These ES cell–derived macrophage data previewed the relative inflammatory potential of each NLRP3 variant and supported the rationale for generating the corresponding humanized mouse lines. Including this supplemental figure documents the basis for that decision and highlights the broader value of ES cell–based assays for anticipating in vivo phenotypes—an approach that will be equally important for the future generation and evaluation of additional NLRP3 variant mouse lines.

.

1. Keller GM, Webb S, and Kennedy M. Hematopoietic Development of ES Cells in Culture. *Methods Mol Med.* 2002;63:209–30.

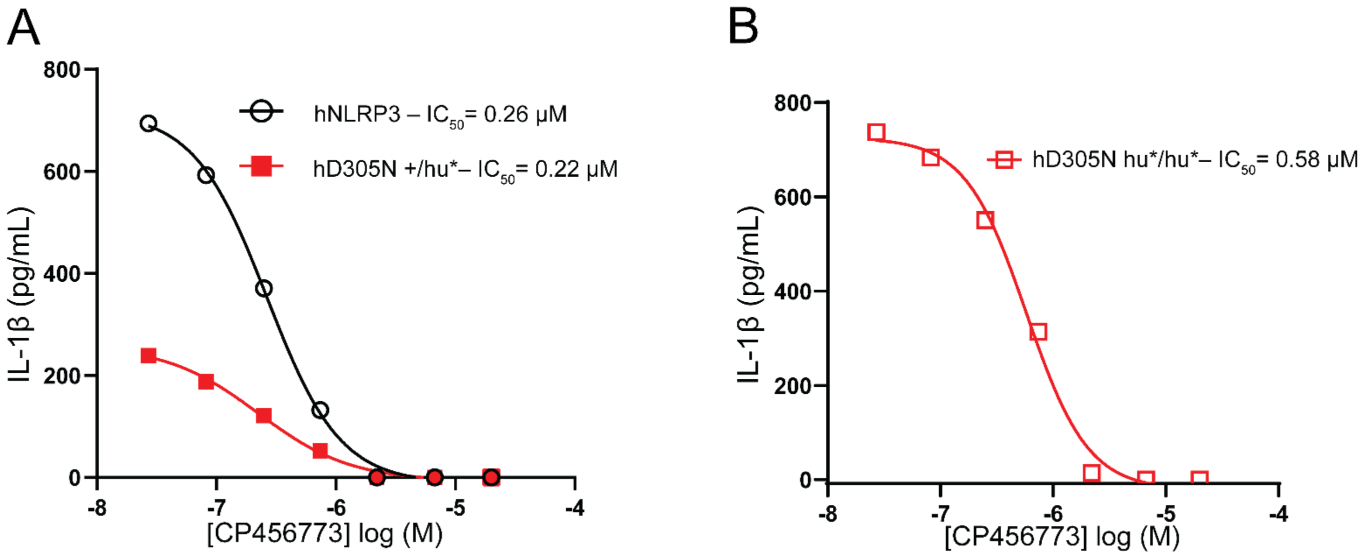

Supplemental Figure 2. Comparison of the sensitivity of blood from heterozygous and homozygous D305N CAPS mice to CP-456,773 inhibition of IL-1β release. Blood was collected from mice of the indicated genotypes and exposed to LPS alone or LPS followed by ATP at 37 °C. (**A**) Blood from mice homozygous for the common *NLRP3* allele (hNLRP3) released IL-1β after LPS/ATP stimulation, and this release was inhibited by CP-456,773 in a concentration-dependent manner. Blood from mice heterozygous (**A**) or homozygous (**B**) for the D305N allele released IL-1β following LPS exposure alone, although the magnitude differed, with higher levels observed in homozygous cultures as expected. IL-1β release from both heterozygous and homozygous D305N blood was inhibited by CP-456,773. Despite the lower total IL-1β release in the heterozygous animals, the observed IC_{50} values were relatively similar across genotypes. The mean IC_{50} for nine independent assays using blood from hNLRP3 mice stimulated with LPS followed by ATP was 0.49 ± 0.05 µM. In comparison, the mean IC_{50} for mice homozygous for the D305N allele, based on four independent experiments, was 0.79 ± 0.10 µM. The mean IC_{50} for three independent experiments using blood from mice heterozygous for the D305N allele was 0.21 ± 0.003 µM.

hNLRP3 hu/hu	D305N hu/hu	D305N+/hu	
IC50 µM	IC50 µM	IC50 µM	
0.49	0.87	0.21	
0.42	0.68	0.22	
0.66	1.04	0.21	
0.44	0.58		
0.66			
0.62			
0.61			
0.26			
0.295			
Mean : 0.49	0.79	0.21	
SEM 0.05	SEM 0.10	SEM 0.003	

Table 1. Main clinical features in patients with cryopyrinopathies (CAPS)

Characteristic	FCAS	MWS	NOMID/ CINCA
**Inheritance**	Autosomal dominant	Autosomal dominant	De Novo<br>Autosomal dominant
**Age of onset**	Birth	Infancy	Birth
**Fever**	+	+	+
**Cutaneous rash**	Cold-induced	+	+
**CNS Inflammation**	Headache	+	+
**Arthritis/bony overgrowth**	–	–/+	+
**Growth retardation**	–	– /+	+
**Frontal bossing, saddleback nose, clubbing**	–	–	+
**Gene mutations**	p.Leu305Pro	p.Asp305Asn	p.Tyr572Cys<br>p.Phe311Ser

FCAS, familial cold autoinflammatory syndrome; MWS, Muckle-Wells; NOMID/CINCA, Neonatal onset multisystem Inflammatory disease/ chronic infantile neurological, cutaneous and articular syndrome.

TABLE 2

Figure 1B

Spleen Wt (Tukey-Kramer test)

	hNLRP3	hL307P	hD305N	hF311S	hY572C
hNLRP3		ns	ns	ns	**
hL307P	ns		ns	ns	*
hD305N	ns	ns		ns	*
hF311S	ns	ns	ns		ns
hY572C	**	*	*	ns	

Figure 1D

IL-1Ra (Tukey-Kramer test)

	hNLRP3	hL307P	hD305N	hF311S	hY572C
hNLRP3		ns	ns	**	****
hL307P	ns		ns	**	****
hD305N	ns	ns		ns	****
hF311S	**	**	ns		*
hY572C	****	****	****	*	

Figure 1E

SAP (Tukey-Kramer test)

	hNLRP3	hL307P	hD305N	hF311S	hY572C
hNLRP3		*	ns	****	****
hL307P	*		ns	ns	***
hD305N	ns	ns		**	****
hF311S	****	ns	**		ns
hY572C	****	***	****	ns	

Figure 1F

IL-1β (Tukey-Kramer test)

	hNLRP3	hL307P	hD305N	hF311S	hY572C
hNLRP3		ns	*	**	**
hL307P	ns		ns	*	*
hD305N	*	ns		ns	ns
hF311S	**	*	ns		ns
hY572C	**	*	ns	ns	

Figure 1G

IL-18 (Tukey-Kramer test)

	hNLRP3	hL307P	hD305N	hF311S	hY572C
hNLRP3		ns	ns	**	ns
hL307P	ns		ns	**	ns
hD305N	ns	ns		ns	ns
hF311S	**	**	ns		ns
hY572C	ns	ns	ns	ns	

Figure 1I

IL-6 (Tukey-Kramer test)

	hNLRP3	hL307P	hD305N	hF311S	hY572C
hNLRP3		ns	ns	**	***
hL307P	ns		ns	*	**
hD305N	ns	ns		ns	ns
hF311S	**	*	ns		ns
hY572C	***	**	ns	ns	

TABLE 3

Figure 2C

PLF-IL-1β (Tukey-Kramer test)

	hNLRP3	hL307P	hD305N	hF311S	hY572C
hNLRP3		ns	ns	***	****
hL307P	ns		ns	***	****
hD305N	ns	ns		*	****
hF311S	***	***	*		**
hY572C	****	****	****	**	

Figure 2E

PGE_2 (Tukey-Kramer test)

	hNLRP3	hL307P	hD305N	hF311S	hY572C
hNLRP3		ns	ns	**	****
hL307P	ns		ns	**	****
hD305N	ns	ns		**	****
hF311S	**	**	**		****
hY572C	****	****	****	****	

Figure 2H

LDH release (Tukey-Kramer test)

	hNLRP3	hL307P	hD305N	hF311S	hY572C
hNLRP3		ns	ns	*	**
hL307P	ns		ns	ns	*
hD305N	ns	ns		ns	ns
hF311S	*	ns	ns		ns
hY572C	**	*	ns	ns	

TABLE 4

Figure 3G - CBC - WBC

CBC-WBC (Tukey-Kramer test)

	hNLRP3	hL307P	hD305N	hF311S	hY572C
hNLRP3		N/A	ns	ns	***
hL307P	N/A		N/A	N/A	N/A
hD305N	ns	N/A		ns	
hF311S	ns	N/A	ns		***
hY572C	***	N/A	***	**	

N/A-evaluation not available

Figure 3G - CBC - neutrophils

CBC-neutrophils (Tukey-Kramer test)

	hNLRP3	hL307P	hD305N	hF311S	hY572C
hNLRP3		N/A	ns	ns	***
hL307P	N/A		N/A	N/A	N/A
hD305N	ns	N/A		ns	***
hF311S	ns	N/A	ns		**
hY572C	***	N/A	***	**	

N/A-evaluation not available

Figure 3G - CBC - lymphocytes

CBC-lymphocytes (Tukey-Kramer test)

	hNLRP3	hL307P	hD305N	hF311S	hY572C
hNLRP3		N/A	ns	ns	**
hL307P	N/A		N/A	N/A	N/A
hD305N	ns	N/A		ns	*
hF311S	ns	N/A	ns		*
hY572C	**	N/A	*	*	

N/A-evaluation not available

Figure 3G - CBC - monoctyes

CBC Monocytes (Tukey-Kramer test)

	hNLRP3	hL307P	hD305N	hF311S	hY572C
hNLRP3		N/A	ns	ns	****
hL307P	N/A		N/A	N/A	N/A
hD305N	ns	N/A		ns	****
hF311S	ns	N/A	ns		****
hY572C	****	N/A	****	****	

N/A-evaluation not available

Comparison of CBC values between hNLRP3 and CAPS genotypes

		Unpaired Welch's t-test	Dunnett's
**Neutrophils**	NLRP3 - D305N	**ns**	**ns**
	NLRP3 - F311S	*****	**ns**
	NLRP3 - Y572C	*****	*******

		Unpaired Welch's t-test	Dunnett's
**Monocytes**	NLRP3 - D305N	******	**ns**
	NLRP3 - F311S	*******	**ns**
	NLRP3 - Y572C	******	********

		Unpaired Welch's t-test	Dunnett's
**Lymphocytes**	NLRP3 - D305N	**ns**	**ns**
	NLRP3 - F311S	*****	**ns**
	NLRP3 - Y527C	**ns**	******

TABLE 5

Figure 4M

Caspase activity (Tukey-Kramer test)

	hNLRP3	Casp1/11$^{-/-}$	hD305N	hF311S	hY572C	hNLRP3-ATP
hNLRP3		**ns**	**ns**	****	****	**ns**
Casp1/11$^{-/-}$	**ns**		**ns**	****	****	**ns**
hD305N	**ns**	**ns**		**	****	**ns**
hF311S	****	****	**		****	****
hY572C	****	****	****	****		****
hNLRP3-ATP	**ns**	**ns**	**ns**	****	****	

Figure 4N

IL-1β (Tukey-Kramer test)

	hNLRP3	Casp1/11$^{-/-}$	hD305N	hF311S	hY572C	hNLRP3-ATP
hNLRP3		**ns**	**	****	****	****
Casp1/11$^{-/-}$	**ns**		**	***	****	****
hD305N	**	**		**ns**	**	***
hF311S	****	***	**ns**		**ns**	*
hY572C	****	****	**	**ns**		**ns**
hNLRP3-ATP	****	****	***	*	**ns**	

TABLE 6

Figure 5C

Total CNS immune cells (Tukey-Kramer test)

	hNLRP3	hL307P	hD305N	hF311S	hY572C
hNLRP3		**ns**	**ns**	**	***
hL307P	**ns**		**ns**	*	**
hD305N	**ns**	**ns**		**ns**	*
hF311S	**	*	**ns**		**ns**
hY572C	***	**	*	**ns**	

Total CNS immune cells (Welch t-test)

	hNLRP3	hL307P	hD305N	hF311S	hY572C
hNLRP3		**ns**	**	**	**
hL307P	**ns**		**	***	*
hD305N	**	**		**ns**	**ns**
hF311S	**	***	**ns**		**ns**
hY572C	**	*	**ns**	**ns**	

Table 7

Reagents, Kits, Antibodies, Equipment, and Software Used in This Study

Category	Reagent / Item	Company	Catalog Number	Notes
**Animal, Animal Housing**	Ventilated cages (Green Line GM500)	Tecniplast	GM500	Individually ventilated cages
	LabDiet PICOLAB Select 5V5R (irradiated)	PMI Nutrition International		Mouse diet
	Nestlets	Ancare		Cage enrichment
	“Mouse house” shelter	Tecniplast		Red-opaque shelter in each cage
	Bed O Cob ¼”	The Andersons		Bedding
Vivarium Temperature	71 ° F / 22 ° C			
Mice	129S6	129S6/SvEvTac, Taconic Bioscience		Humanized mice are co-isogenic with this strain
**Chemicals & General Reagents**				
	LPS (E. coli 55:B5)	Sigma-Aldrich	L4005	In vivo and vivo studies
	Heparin 1000U/ml	Sargent Pharmaceuticals	NCD25021-400-10	Blood collection
	CP-456,773	Sigma-Aldrich	PZ0280	NLRP3 inhibitor
	TRI reagent	Molecular Research Center	TR118	RNA isolation
	ATP	Sigma-Aldrich	A3377	
	Percoll (isotonic)	Sigma-Aldrich	P1644	Density gradient
	TRI reagent	Molecular Research Center	TR118	RNA isolation
**Cell Isolation/Culture**				
	Fetal Bovine Serum	VWR	97068–085	
	HEPES	Corning	25-060-CI	
	βME	Sigma-Aldrich	M3148	
	L glutamine	Gibco (ThermoFisher)	25030-081	
	Hank’s Balanced Salt Solution	Gibco (ThermoFisher)	14175–095	Without Ca^{2+}/Mg^{2+}
	Dulbecco’s Phosphate Buffered Saline	Sigma-Aldrich	D8537	
	Penicillin-Streptomycin (10,000 U/mL)	Gibco (ThermoFisher)	15140-122	
	RPMI 1640	Gibco (ThermoFisher)	11875–093	
**Enzymes/inhibitors**				
	Collagenase Type 4	Worthington Biochem (ThermoFisher)	LS004186	
	DNase I	Sigma-Aldrich	DN25	Brain tissue dissociation

	cOmplete ™ Protease Inhibitor Cocktail	Roche Millipore-Sigma	11697498001	Preparation of tissue homogenates
**Kits & Assays**	LDH Cytotoxicity Detection Kit	Sigma-Aldrich	4744926001	LDH assay
	Caspase-Glo 1 Inflammasome Assay Kit	Promega	G9951	Caspase-1 activity
	High-Capacity cDNA Reverse Transcription Kit	Applied Biosystems (Thermo-Fisher)	4368814	cDNA synthesis
	qPCRBIO Probe Blue Mix Lo-ROX - universal probe kit	Genesee	17-514B	qPCR
**ELISA Kits**				
	IL-1β ELISA	R&D Systems	DY401	
	TNF-α ELISA	Invitrogen Thermo Fisher	88-7324-88	
	IL-6 ELISA	Invitrogen Thermo/Fisher	88-7004-88	
	IL-18 ELISA	Invitrogen ThermoFisher	BMS618-3	
	IL-1ra/IL-1F13	R&D Systems	MRA00	
	CXCL1/KC DuoSet	R&D	DY453	
	Serum amyloid P ELISA	Immunology Consultants Laboratory	E-90SAP	
	PGE_2	ENZO	ADI-901-001	
**Antibodies**	Anti-CD11b (flow cytometry)	BioLegend	101237	
	Anti-CD45 (flow cytometry)	BioLegend	103107	
	Anti-CD16/32 Fc block	BioLegend	101302	Fc receptor blocking
**Plastics / Labware**	White 96-well assay plates	Falcon	353596	Luminescence assays
**Equipment**	BioTek Synergy 2 Plate Reader	BioTek		Luminescence
	FastPrep-24 126-48 instrument	MP Biomedicals	116004500	Homogenization
	Attune NxT Flow Cytometer	Thermo Fisher		Flow cytometry
	QuantStudio 6 Flex qPCR system	Applied Biosystems		qPCR
	Physitemp rectal probe	Physitemp Instruments		Temperature measurement
	gentleMACS dissociator	Miltenyi Biotec		Dissociation of soft tissues
**Software**				
	GraphPad Prism	GraphPad Software	v10.6.1	Statistics
	Attune NxT Software	Thermo Fisher		Flow cytometry acquisition
	FlowJo	FlowJo LLC Ashland, Oregon	v10.6.1	Cytometry analysis

**Gene expression assays**		
TaqMan™ Gene Expression Assay (FAM), Mouse *Il6*	Thermofisher	Mm00446190_m1
TaqMan™ Gene Expression Assay (FAM), Human *NLRP3*	Thermofisher	Hs00918082_m1
TaqMan™ Gene Expression Assay (FAM), Mouse *Chil3*	Thermofisher	Mm00657889_mH
TaqMan™ Gene Expression Assay (FAM), Mouse *Il1rn*	Thermofisher	Mm00446186_m1
TaqMan™ Gene Expression Assay (FAM), Mouse *Tnf*	Thermofisher	Mm00443258_m1
TaqMan™ Gene Expression Assay (FAM), Mouse *Gfap*	Thermofisher	Mm01253033_m1
TaqMan™ Gene Expression Assay (FAM), Mouse *Il1b*	Thermofisher	Mm00434228_m1
TaqMan™ Gene Expression Assay (FAM), Mouse *Saa1/2*	Thermofisher	Mm04208126_mH
TaqMan™ Gene Expression Assay (FAM), Mouse *Mpo*	Thermofisher	Mm00447886_m1
TaqMan™ Gene Expression Assay (FAM),Human 18s RNA	Thermofisher	H5999999-S1
